# How Task Set and Task Switching Modulate Perceptual Processes: Is Recognition of Facial Emotion an Exception?

**DOI:** 10.5334/joc.179

**Published:** 2021-08-05

**Authors:** Heike Elchlepp, Stephen Monsell, Aureliu Lavric

**Affiliations:** 1 Department of Psychology, University of Exeter, UK

**Keywords:** Cognitive Control, Attention, Face perception, EEG, Emotion and cognition

## Abstract

In Part 1 we review task-switching and other studies showing that, even with time for preparation, participants’ ability to shift attention to a relevant attribute or object before the stimulus onset is limited: there is a ‘residual cost’. In particular, several brain potential markers of perceptual encoding are delayed on task-switch trials, compared to task-repeat trials that require attention to the same attribute as before. Such effects have been documented even for a process often considered ‘automatic’ – visual word recognition: ERP markers of word frequency and word/nonword status are (1) delayed when the word recognition task follows a judgement of a perceptual property compared to repeating the lexical task, and (2) strongly attenuated during the perceptual judgements. Thus, even lexical access seems influenced by the task/attentional set.

In Part 2, we report in detail a demonstration of what seems to be a special case, where task-set and a task switch have no such effect on perceptual encoding. Participants saw an outline letter superimposed on a face expressing neutral or negative emotion, and were auditorily cued to categorise the letter as vowel/consonant, or the face as emotional/neutral. ERPs exhibited a robust emotional-neutral difference (Emotional Expression Effect) no smaller or later when switching to the face task than when repeating it; in the first half of its time-course it did not vary with the task at all. The initial encoding of the valence of a fixated facial emotional expression appears to be involuntary and invariant, whatever the endogenous task/attentional set.

To accomplish multiple goals in a given time period, we may attempt concurrent performance of multiple tasks. More often, perhaps, we rapidly switch between tasks. To investigate how the cognitive system adapts its processing to such changes in its goals, we can train participants on simple tasks and then require them to switch frequently among them (see Kiesel et al., 2010; Monsell, 2015, 2017; Vandierendonck et al., 2010, for reviews). Perhaps unsurprisingly, a switch of tasks results in impaired performance, a ‘switch cost’. Given time to prepare for a task switch (e.g. by increasing the interval between task cue and stimulus) participants can reduce the switch cost, suggesting proactive reconfiguration of task set (e.g., Lavric, Mizon & Monsell, 2008; Vant Wout, Lavric, & Monsell, 2013). Also intriguing is the non-trivial fraction of the switch cost which remains even when the participant has both the incentive and ample time to prepare for the switch (e.g., Rogers & Monsell, 1995, Meiran, 1996; Nieuwenhuis & Monsell, 2002). Until recently, a widely-held view was that this ‘residual’ (asymptotic) switch cost reflects interference largely from persistence or reactivation of the stimulus-response (S-R) mappings associated with the competing task-set(s) (see Elchlepp, Lavric & Monsell, 2015, for a detailed review). Yet, in many (if not most) task-switching studies the different tasks require attention to different perceptual attributes or objects (e.g., letters vs. digits, colours vs. shapes, etc.). To what extent do the costs of switching between tasks derive from difficulty in shifting attention between attributes and objects, and hence to retarded perceptual processing of the input?

This article has two parts. In Part 1, we first review (Section 1a) recent studies suggesting that, for a wide range of perceptual attributes and objects, participants’ ability to shift attention to the relevant attribute or object before the next stimulus onset is limited even when adequate time is available: there is an unavoidable residual cost attributable to less efficient perceptual processing of the stimulus on the switch trial. Such observations have, we point out in section 1b, consequences for claims that certain cognitive processes are ‘automatic’ – in the sense of proceeding involuntarily (without intention), and/or ‘invariantly’ regardless of the task. A paradigm case is visual word recognition. We review data from the task-switching paradigm showing that even word recognition is slowed and suppressed when attention is either focused on, or has just been focused on, a non-lexical attribute of words. But is it generally true that all candidates for automaticity of perceptual processing, examined in this way, will turn out to be modulated by task and task-switching? In the second part of the article, we present new data suggesting an interesting exception: recognition of emotional expression in faces. We present EEG data suggesting that, with adequate opportunity for pre-stimulus preparation, discrimination of emotional and non-emotional faces proceeds without delay and, in its early stages, invariantly, even when attention is, or has just been, focused on a different attribute in the same location.

## 1. Modulation of perceptual processing by task-readiness

### 1.1 Attention switching incurs a cost, even with time to prepare

One source of evidence is represented by experiments where everything about the task remains the same from trial to trial except the target of attention, which is cued with adequate time to prepare. In the visual search literature, several studies have found that switching between visual dimensions, such as colour and form, is associated with a cost, especially when the target of a search is a conjunction of perceptual features (Weidner & Müller, 2009), but also in single-feature (‘pop-out’) search (Müller, Reimann, & Krummenacher, 2003) – and even in ‘compound’ search where the response is dictated by an orthogonal property of the singleton target, e.g. one responds to the orientation of a grating inside the colour target (Töllner, Gramann, Müller, Kiss, & Eimer, 2008). However, because these studies did not systematically examine preparation, it is not clear that the ‘dimension change’ costs they revealed were asymptotic.

Several studies have clarified this by adapting the task-cueing paradigm (Meiran, 1996; 2014) to examine changes of ‘attentional set’ – switches in the attentional component of the task-set – whilst other task-set components (in particular S-R rules) were kept constant. These studies have either compared shorter and longer preparation intervals (cue-stimulus intervals, CSIs) or used quite long CSIs, hence, at least under some conditions, they examined prepared switches. Their results show that whether or not switching the attentional component alone results in a residual switch cost depends on the type of perceptual attribute under scrutiny.

*Perceptual dimensions*. Meiran and Marciano (2002) asked participants to make same-different judgements on pairs of geometric shapes varying over 4 dimensions (shape, size, tilt and fill); a verbal cue specified the target dimension. Generous increases in CSI (to ~1.5 s and even ~3 s) did not reduce a substantial ‘dimension switch cost’. In contrast, the authors found that switch costs resulting from reversals of S-R mappings reduced substantially with preparation. Rushworth, Passingham, and Nobre (2005) asked participants to identify a symbol in one of two coloured geometric figures. A cue presented at CSI = 2000 ms specified the figure by either colour or shape; the S-R rules remained constant. There was a substantial dimension change cost despite the long CSI.

*Features (values) on the same dimension*: Lien, Ruthruff, and Johnston (2010) asked participants to select, in an array of coloured digits, the one whose colour was specified in advance (CSI = 1350 ms) by a transparent letter cue (e.g., ‘R’ specified red), and classify the selected digit by parity (some participants) or magnitude (other participants). Importantly, 50 ms before the stimulus array, an uninformative ‘capture cue’ was presented which matched (or not) the target digit in colour and location. Based on previous research (Folk, Remington, & Johnston, 1992), the authors expected to find faster responses to targets preceded by capture cues at the same locations – but only if the colour of the capture cue matched the target colour. The results indeed revealed this “contingent capture” effect. Its magnitude was not reduced when the target colour switched, and there was no evidence of capture by the previously (but no longer) relevant colour, suggesting that in this case – shifting attention between features within the colour dimension – preparation is highly effective.

*Sensory modalities or spatial locations*. These appear to be two further cases where effective proactive shifts of attention have been reported. Lukas, Philipp, and Koch (2010) investigated shifts of attention between vision and hearing, by presenting compounds made of a tone and a visual stimulus (both lateralised), cueing the target modality and requiring a left-right judgement. The ‘modality switch cost’ was reduced from 123 ms at CSI = 200 ms to only 22 ms when CSI = 1000 ms. Fintor, Stephan, and Koch (2019) used a similar paradigm but required vocal responses for auditory targets and manual responses for the visual targets –their ‘modality switch cost’ was altogether eliminated by preparation. Finally, Logan (2005) investigated shifts of spatial attention by presenting arrays of four letters; participants had to report the presence of a vowel in any of two locations, each cued exogenously by a black dot at a variable CSI. There was a spatial attention shift cost when the CSI was short (≤300 ms), but a CSI of 400 ms seemed sufficient to eliminate it.

*Speakers in a multitalker environment*. Koch and colleagues (e.g., Koch, Lawo, Fels, & Vorländer, 2011; Lawo, Fels, Oberem, & Koch, 2014) presented dichotically male-female pairs of voices (each saying a number) and asked participants to classify by magnitude the number spoken by the target voice cued by either gender or side. A large cost of switching the target voice was not reduced by increasing the CSI. However, preparation did reduce the “voice” switch cost in two experiments of our own (Monsell, Lavric, Strivens & Paul, 2019). They were designed to maximise the effects, and detection of, preparation through the use of a relatively low proportion of switches and response congruent trials, only two central (non-lateralised) voices throughout the experiment, and monetary incentives for good performance. However, we too found that even a generous preparation interval (1.4 s) did not eliminate the ‘voice switch cost’, leaving a very substantial asymptotic residual component.

To summarise, evidence from studies where ‘attentional set’ is the only component of the task-set to change, suggests that some shifts of attention (between modalities, locations, or features) can be executed effectively in advance of the stimulus if the preparation interval permits. But, this does not seem to be the case for switching attention between visual dimensions, or between voices in a multi-talker situation. However, this is not the whole story. What happens if ‘attentional set’ is not the only component to change during a switch? Are the more effortless attentional shifts (e.g., of spatial attention) equally effective when other task-set components, such as the S-R rules, also change, as is typically the case in task-switching studies?

*Attentional shifts when other task-set components also switch*. Recently we used eye-tracking to address this question with respect to spatial attention (Longman, Lavric, & Monsell, 2013; 2016; 2017; Longman, Lavric, Munteanu, & Monsell, 2014). On each trial, three digits were displayed simultaneously at predetermined locations. A central arbitrary cue (e.g. a letter) specified one of three number classification tasks that had to be performed. Crucially, each task was consistently linked to its own location on the screen (balanced over participants), so that the cue also specified which of the three numbers they had to (spatially) select for classification. In one study (Longman et al., 2014), we compared this task-switching condition to a control condition where the same central cue specified the target location, but only one number classification task had to be performed irrespective of location, for the entire experiment. Eye-tracking revealed only a modest delay in fixating the relevant location on switch trials in the control condition (consistent with the studies reviewed above), but in the task-cueing condition the switch-induced delay in fixating the relevant location was much larger (by an order of magnitude). Furthermore, in the task- (but not the location-) cueing condition switching increased the tendency to fixate the previous (no longer relevant) location, even when there was ample opportunity for preparation (CSI ~ 1.4 s) – evidence of ‘attentional inertia’.

Changing multiple task-set parameters might have this dramatic effect – causing delays and inertia in shifting spatial attention, because in the task-switching condition in Longman et al. (2014), spatial attention became somehow ‘coupled’ with other components of the task-set, so that reconfiguration delays (and inertia) in those components led to attentional delays and inertia. Consistent with this account, when Longman et al. (2016) replaced the task cues used in their earlier study with more exogenous spatial cues – central arrows and location words (e.g., “top”), this markedly reduced task-switch-induced delays in orienting and essentially eliminated attentional inertia, even when CSI was short. Thus, proactive shifts of spatial attention accompanying a task switch can be very effective, but only if attention is ‘decoupled’ from the rest of the task-set and given high priority (e.g., by exogenous cuing). In another study (Longman et al., 2017), we found that attentional inertia (though not the delay induced by a task switch) could also be overcome if the participant was given control of the preparation interval – by displaying the number array only when participants’ eyes left the location of the task cue.

Recent eye-tracking research has also examined attentional switches to dimensions when other task-set components also change. Mayr and colleagues (Mayr, Kuhns, & Hubbard, 2014; Kikumoto, Hubbard, & Mayr, 2016) displayed three vertical bars in a triangular array, one different in colour from the other two and one interrupted by a gap; the locations of these singletons varied unpredictably. Participants responded either to the colour of the colour singleton or the position of the gap in the gap singleton. A verbal cue specified the relevant task (and perceptual dimension). Consistent with dimension-switching studies reviewed above (and with our eye-tracking studies of spatial attention), they found substantial delays in selecting the relevant dimension when the task (and the associated dimension) changed. However, contrary to Meiran and Marciano’s (2002) study, they also found that increasing the CSI reduced the switch-related delay in dimension selection considerably – though it did not eliminate it. Finally, in a recent EEG study (Elchlepp, Best, Lavric, & Monsell, 2017), we asked participants to classify a letter as vowel/consonant, or its colour as “warm”/“cold”. A prepared switch to the colour task (relative to repeating it) delayed, by 17 ms, an ERP marker of colour processing starting at ~150 ms following stimulus onset. All the above evidence indicates that on a task-switch trial, even after preparation, the re-allocation of spatial and non-spatial attention can be limited/prolonged/delayed; this can be an important source of the residual cost of a task switch.

### 1.2 Automaticity of processing? Word recognition as the paradigm case

Experiments that require switches between tasks with different targets of attention can illuminate long-running debates over the extent to which certain cognitive processes are ‘automatic’. A classic claim is that visual word identification up to and including semantic activation is, in a literate person, ‘automatic’ in the sense of involuntary^1^ (Posner & Snyder, 1975). The most compelling evidence that words are read in spite of intention is the classic Stroop effect – naming the colour in which a word is printed is slowed when it is an incongruent colour word (MacLeod, 1991), and variants: the ‘semantic Stroop effect’ — interference from colour-associated words (e.g., SKY) indicating that the word has activated its meaning (Neely & Kavan, 2001), and interference from a pronounceable letter string versus a string of Xs or false font string (Monsell, Taylor, & Waters, 2001; Kinoshita, De Wit, & Norris, 2017) indicating that the letter string has activated its pronunciation. Another classic piece of evidence is the observation of ‘automatic’ semantic priming at short inter-stimulus intervals, while priming at long intervals is modulated by expectation (Neely, 1977).

We need first to limit consideration of this issue to conditions in which the focus and distribution of spatial attention are appropriate for reading: i.e. the letter string is within the attentional spotlight, and attention is distributed over all the letters (cf. Neely & Kavan, 2001). Although naming of the colour of a patch can be interfered with by a spatially separate word (e.g., Kahneman & Chajczyk, 1983), there is minimal interference if the focus of attention on the colour-named stimulus is adequately controlled (Robidoux & Besner, 2015). If just one letter in the word is coloured, this can eliminate or severely reduce the Stroop effect (Besner, Stolz, & Boutilier, 1997; Besner et al., 2016, for review). Augustinova and Ferrand (2014) have claimed that the semantic Stroop effect – a purer test of semantic activation (Neely & Kavan, 2001), is not eliminated by restricting the colour to a single letter, but Labuschagne & Besner (2015) found that it is if the letters are spaced and attention directed to the letter.

With this qualification – appropriate distribution of spatial attention – in mind, we now consider evidence suggesting that the activation of meaning or pronunciation by a printed word is not invariant but strongly influenced by the task-set adopted by the participant – i.e. whether the participant intends to read (cf. Kiefer & Martens, 2010). One line of research has asked whether semantic priming (usually of lexical decision) by a preceding or accompanying prime word is modulated by the task applied to the prime word. The typical finding (Maxfield, 1997) is robust semantic priming when the participant must name the prime word, but little or none when the prime task is to search the prime word for a letter. However, the task of letter search violates our criterion of appropriate spatial attention. Another example of modulation of word reading by task-set was observed by Kinoshita et al., (2017), who found that a gradient of interference found over words, pronounceable pseudowords, and consonant strings, when participants named the colour, disappeared when key presses were used to indicate the colour, though all these stimuli still generated interference relative to XXXX strings. They concluded that the involuntary generation of a letter string’s pronunciation is dependent on the adoption of a naming task-set.

Trial to trial manipulation of task-set through a task-switching experiment seems an obvious way of addressing the question, with one task requiring lexical access, and the other requiring attention to a non-lexical property of the letter string. If lexical access is amplified, or partially suppressed, by whether the participant intends to read or to respond to a non-lexical property of the stimulus word, then measures of word recognition should be modulated by switching between the reading task and the non-lexical task.

Kiefer and Martens (2010, Exp. 1) examined priming of lexical decision by a masked semantic prime, using ERPs. Preceding each priming test, participants either classified a word semantically (living/non-living), or performed a task requiring inspection of the first and last letters of the word. With 200 ms between the response to the first task and the invisible prime, both RT and the N400 showed robust semantic priming when the first task was a semantic decision, but a weaker effect when the first task was a perceptual task; this pattern was reversed at a long (800 ms) interval because, they suggested, there was now sufficient time to suppress the first task set and prepare for the lexical decision task. Vachon and Jolicoeur (2011) examined the effect of a task switch in an attentional blink paradigm, with rapid presentation, following a context word, of a sequence of character strings, of which two were word-containing targets such as 3XXPIGXX6. Participants decided whether the word in the second target was semantically related to the context word. With a short lag between the targets, accuracy and the amplitude and latency of the N400 were substantially modulated by whether the task performed on the first target was the same semantic judgement, or the perceptual task of deciding whether the terminal digits matched. Vachon and Jolicoeur (2012) observed similar attenuation of N400 in a PRP experiment using the same tasks and stimulus onsets separated by 100–700 ms. However, the perceptual tasks in all these studies required attention to just the terminal characters of the string; this may distribute spatial attention in a way non-optimal for recognition of the word.^2^

In an EEG study of our own (Elchlepp et al., 2015), participants classified a letter string containing red and blue letters with respect to either a lexical property (semantic category in one experiment, word/nonword in another), or a perceptual property – the symmetry of the distribution of colours over the letters – a task chosen to distribute spatial attention over the whole string. There was an 800 ms interval between task cue and stimulus, and evidence that participants used it for task-set preparation. On the lexical task trials, brain potentials indicative of lexical processing (a word-nonword difference, from ~180 ms post-cue, and a lexical frequency effect, from ~200 ms) were substantially delayed when the perceptual rather than the lexical task was performed on the previous trial. Thus, attention to lexical versus perceptual properties of a letter string provides another case where attentional inertia contributes to the residual cost of a task switch. On the perceptual task trials both lexico-semantic brain potentials were still present – indicating involuntary activation of lexical representations – but they were strongly attenuated and delayed relative to the lexical task trials. It seems, therefore, that early stages of visual word recognition are subject to top-down control of task-set: although involuntary, lexical activation is far from invariant.

One other fragment of task-switching evidence merits a mention. Approximate additivity between the effect of varying stimulus contrast and the effect of a switch between lexical and non-lexical tasks was reported by Besner and Care (2003) for switching between nonword reading and classification as upper/lower case, and by Oriet and Jolicoeur (2003) for classifying a digit^3^ by magnitude or parity; the latter study also showed additivity of the contrast effect with that of preparation interval. The authors of both studies concluded that perceptual processing must be suspended until a task-set reconfiguration process has been completed. However, a more straightforward additive-factors interpretation is that the duration of a very early stage of stimulus processing, at which contrast has its effect, is simply uninfluenced by whether the task repeats or switches, or by the state of task-set preparation; it is only later stages of perceptual encoding whose duration are influenced by the adoption of a reading or non-lexical task set.

In summary, evidence from task switching experiments yields a clear conclusion: given an appropriate spread of spatial attention over the letter string, some degree of lexical access may be involuntary for familiar words, but it is certainly not invariant in relation to either the current or the immediately preceding task-set. Somewhere between the very early analysis of contours and activation of lexical representations, processing is impaired when the previous task performed was not a reading task, even with time for preparation of the reading task-set. When the current task requires attention to non-lexical attributes, lexical and semantic activation is markedly attenuated.

## 2. Effects of a task switch on the recognition of emotional expression

We have reviewed two kinds of experiment: those in which only attention has to shift from trial to trial, and those in which attention is one component of the task-set that has to change. Both kinds of experiment indicate that, for some attributes or objects of attention, the efficacy with which participants can proactively reset attentional parameters from trial to trial, even with ample time to prepare, is limited. The allocation of spatial attention may be delayed, and the re-allocation of attention to the relevant dimension or complex attribute such as voice or lexical content is initially non-optimal (subject to attentional inertia) with the result that perceptual processes are prolonged on switch trials. Now we turn to consider the efficacy of shifting attention to and from another important class of stimulus – faces.

A priori, face perception seems even more likely to be “automatic”, in the sense of invariant, than word recognition. Rapid recognition of facial identity, gender, age and expression, even when they are not the current target of attention, has an obvious adaptive advantage, and people arguably have even more practice, and therefore expertise, on “reading” faces than on reading words. At least some aspects of face processing do appear to be involuntary. Some persuasive evidence comes from a task-switching fMRI study. Yeung, Nystrom, Aronson, and Cohen (2006) had participants classify faces by gender or words as bi-syllabic or not. The two tasks were first performed in separate blocks to obtain “localiser scans” – brain activations diagnostic of each task. In the critical (test) phase, participants were then presented with word-face compounds, in short (4-trial) runs on either the gender task or the word task cued at the beginning of each run by a central colour patch. Yeung et al. found fusiform gyrus activity (which localiser scans had associated with face processing) not only on the face task trials (as one would expect), but also on the word task trials, especially just after a switch from the face task. Furthermore, the difference in face-related activity on switches to, versus repetitions of, the word task predicted the RT cost of switching to the word task. However, although this indicates involuntary face perception when the word task was cued, it does not demonstrate invariance: in fact, face-related fusiform activity was somewhat reduced on switches to (compared to repetitions of) the face task and it was strongly attenuated during the word task relative to the face task.

We now report an experiment exploring another plausible candidate for involuntariness – and possibly invariance – in face processing: recognition of emotional expression. Importantly, this has a well-documented ERP correlate – the Emotional Expression Effect (EEE, see Eimer & Holmes, 2007, for a review) – a protracted positive-polarity deflection with a central-frontal scalp distribution induced, from ~150 ms until ~300–400 ms following the stimulus onset, by a photograph of a face expressing an emotion relative to a face with a neutral expression. Holmes et al. (2006) investigated the EEE by presenting a single face (fearful or neutral) foveally, flanked by a pair of vertical lines identical or different in length. In one half of the experiment, the face was task-relevant: participants had to attend to it to detect immediate repetitions of the same face on successive trials. In the other half, the face was task-irrelevant: participants had to detect immediate repetitions of the pair of lines. Holmes and colleagues found an EEE during the line task at 160–220 ms following the onset of the face-lines compound, and it was statistically indistinguishable from the EEE in the face task blocks. In its later portion (at 220–300 ms following stimulus onset) the EEE was only detectable in the face task. These results suggest that the EEE is involuntary (it is observed when attention to facial expression is irrelevant, even potentially detrimental, to task performance) and, at least in its early stages, invariant – its amplitude did not reduce in the line judgement task. Other evidence that the EEE can be observed in the absence of the intention to process, or indeed awareness of, a face stimulus comes from a study by Eimer and Kiss (2008) who used subliminal presentation, along with carefully designed sensitive measures of conscious awareness. They found a significant EEE in the absence of conscious awareness.

**Figure 1:**
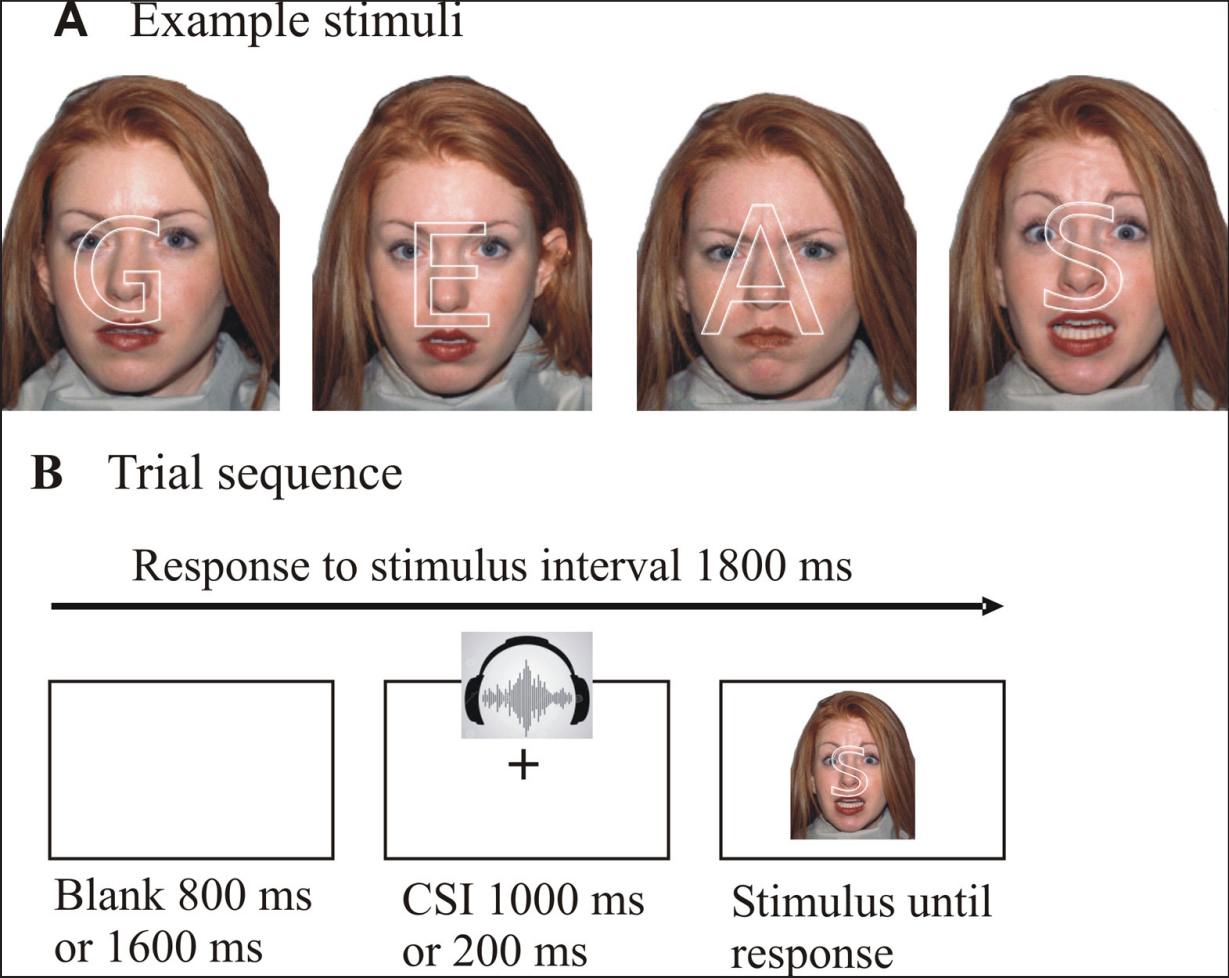
Illustration of stimuli (A), comprising the face of one individual with two different neutral expressions (with letters “G” and “E” superimposed), an angry expression (with “A” superimposed) and a fearful expression (with “S” superimposed). The sequence of displays on one trial is shown in panel B.

**Figure 2:**
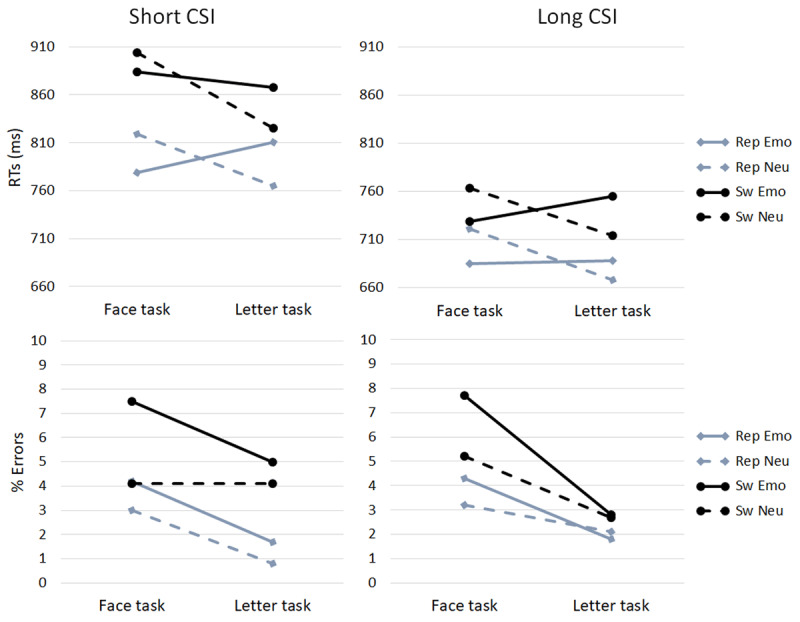
RTs and error rates by CSI, task, switch/repeat and emotional expression (“Emo”: emotional expressions; “Neu”: neutral expressions).

In the present study we applied to the EEE the same basic method and inferential logic we have previously employed in our investigations (described above) of switching attention between lexical and non-lexical attributes of visual words (Elchlepp et al., 2015) and between the colour and shape of letters (Elchlepp et al., 2017). We asked our primary question – does a shift of attention to facial expression from a non-face property of the stimulus result in a processing cost, even when there is opportunity for preparation? – by examining the EEE in response to face-letter compounds, in which participants were asked to categorise the letter as vowel/consonant, or the facial expression as emotional/neutral. Holmes, Vuilleumier and Eimer (2003) have shown that when task-irrelevant faces are presented extra-foveally as part of an array also containing non-face stimuli, and participants are required to attend to the non-face stimuli, the EEE is abolished, suggesting that the cortical component of recognition of facial expression (which the EEE is thought to reflect) requires spatial attention and, possibly, fixation (cf. word reading, as discussed in Section 1.2). Hence, we sought to minimise the face-letter spatial separability by: (1) superimposing relatively large transparent letter outlines on faces (see ***Figure 1***), and (2) varying the exact size of the letter and its location on the face, whilst ensuring that the letter was always superimposed on a substantial portion of the eyes-nose-mouth region of the face.

Our reasoning (analogous to that of Elchlepp et al., 2015 and Elchlepp et al., 2017) is that if perceptual encoding of emotional expression is independent of task-set we should find its electrophysiological ‘signature’ (the EEE) uninfluenced by switching from the letter task. Our analyses will also further interrogate the “automaticity” of recognition of emotional expression. Strong evidence that it is both involuntary and invariant would be the equivalence of EEE (at least its earliest portion) in the face and letter tasks, as previously found by Holmes et al. (2006). Conversely, if processing of emotional expression is modulated by endogenous task-set control in the same way as that of the perceptual attributes discussed above, then the EEE should be delayed/attenuated on a face task trial that follows a letter task trial and strongly suppressed in the letter task.

## Method

### Participants

Twenty right-handed students from the University of Exeter (17 female, 3 male) aged between 18 and 27 (M = 19, SD = 2) gave informed written consent for participating in the study run in accordance with the guidelines set by the University of Exeter School of Psychology Ethics Committee, and received course credits for participation plus a monetary performance-related bonus (maximum £4, see Procedure).

### Stimuli and Procedure

Colour photographs of 20 individuals’ faces (ten males and ten females) were selected from the NimStim database of facial expressions (*http://macbrain.org/resources.htm*, Tottenham et al., 2009). There were 80 photos in total, four of each person, two for each kind of facial expression (emotional or neutral), one with mouth open, one with mouth closed (see examples in ***Figure 1***), to avoid the open/closed mouth being diagnostic of the facial expression. We used the emotional expressions of anger or fear (one of each for each person), because these emotions were previously found to elicit the largest EEE (emotional-neutral difference, Eimer & Holmes, 2002; 2007; Eimer & Kiss 2007). Each photo was displayed so as to subtend 4° of visual angle horizontally and 5° vertically. On each photo one of 8 letters (half vowels, half consonants) was superimposed: A, E, O, U, V, S, N, or G. A thin, unfilled, outline of the letter displayed in white, upper case Lucinda Console font covered the central region of the face, including the eyes and mouth (see ***Figure 1***). The letter’s average size was chosen to roughly match the area of the face that is attended to when judging emotional expression (this was guided by the distribution of fixations during face identification from a previous eye-tracking study in our laboratory, Longman et al., 2013). The size of the letter varied slightly between 1.6° and 2.1° (for illustration, see the variable letter size in ***Figure 1***) to match the variability in area attended for face processing and to make it harder to anticipate which facial feature the letter will encompass. The aim was to match the spread and variability of the focus of visual attention approximately in the letter and face tasks to minimize any contribution of spatial attention switching or zooming to the switch cost.

On each trial, participants had to perform one of two tasks. In the ‘face task’, participants indicated whether the facial expression was emotional or neutral by pressing the left or right arrow key (respectively) with the corresponding index finger. In the ‘letter’ task, participants pressed the left key for vowels and the right key for consonants. The sequence of tasks was unpredictable, with task identity indicated on every trial by one of four auditory cues all with a duration of 320 ms: “emotion” and “feeling” for the face task, and “letter” and “symbol” for the letter task. The task changed on a random third of the trials: p (task switch) = 0.33; previous research (cf. Monsell & Mizon, 2006) has suggested that when p(task switch) is higher (≥ 0.5), participants tend to anticipate (and prepare for) a switch before the task cue is presented, which reduces the sensitivity of the switch vs. repeat comparison. The cue changed from trial to trial even on task repetition trials to unconfound task from cue changes (cf. Monsell & Mizon, 2006). There were 15 blocks of 80 trials each plus a start-up trial which was not analyzed; in 12 of the blocks the CSI was 1000 ms, in the remaining 3 blocks (20% of trials) it was 200 ms (in the short CSI the auditory cue thus carried over into the stimulus presentation by 120 ms). Short CSI blocks were interspersed among long CSI blocks in positions: 1, 6, 11 (i.e. SLLLLSLLLLSLLLL); 2, 7, and 12; 3, 8, and 13; 4, 9, and 14; or 5, 10 and 15 – each of these orders used for four participants. The stimulus was displayed until a response was made, after which there was an 1800 ms interval until the next stimulus except after an error – when “Error” was displayed for an additional 1200 ms.

The assignment of individual face × expression combinations (20 faces with emotional and 20 faces with neutral expression) to the combination of task × switch/repeat was counterbalanced across 40*6 = 240 trials (i.e., three blocks). If an even-numbered participant saw a particular face with the mouth open, odd-numbered participants saw that face with the mouth closed, and vice versa.

The EEG session was preceded by a practice session comprising 4 single-task blocks of 24 trials each (two blocks per task) and 2 blocks of 48 trials of task switching. Following practice, the EEG was acquired for the entire duration of the testing. Participants were instructed to use the cue to prepare for the upcoming task. To encourage effective preparation, an incentive scheme was employed: A score (mean RT/10 + errors*5) was computed for each block and a bonus payment was made for blocks on which the score was lower than a running average of the previous blocks with the same CSI.

### EEG/ERP

The EEG was sampled continuously at 500 Hz with a bandpass of 0.016–100 Hz, the reference at Cz and the ground at AFz using 64 Ag/AgCl active electrodes (ActiCap, Brain Products, Munich, Germany) connected to BrainAmp amplifiers (Brain Products, Munich, Germany). There were 62 electrodes on the scalp in an extended 10–20 configuration and one on each earlobe. Electrode locations were adjusted using a CMS ultrasound digitizer (Zebris Medical, Isny, Germany) and their impedances kept below 10kΩ. The EEG was filtered offline with a 20 Hz low-pass filter (24 dB/oct) and re-referenced to the linked ears. The experiment was designed to examine the potential delay in attention to facial expression on prepared switch trials (cf. Elchlepp et al., 2015; Elchlepp et al., 2017). Hence, just enough short CSI trials were included to confirm effective preparation in RT and error data (as indexed by the reduction in switch cost with an increase in CSI), but this number of short CSI trials (20 for each switch × task × facial expression analysis cell) was inadequate for ERP analyses. Hence, ERPs were obtained only for the long CSI for which there was a much (5 ×) larger number of trials available.

The EEG was first segmented into 1600 ms epochs time-locked to the cue and baseline-corrected relative to the average amplitude of the 100 ms preceding the cue. Subsequently, from these epochs shorter segments were obtained starting from 100 ms preceding the stimulus to 600 ms following stimulus onset. As in our previous studies of switch-induced attentional delays (Elchlepp et al., 2015; Elchlepp et al., 2017), we used a pre-cue baseline instead of a pre-stimulus baseline because the above studies and other task-switching and language-switching studies from our laboratory (Elchlepp, Lavric, Mizon, & Monsell, 2012; Elchlepp, Lavric, Chambers, & Verbruggen, 2016; Lavric et al., 2008; Lavric, Clapp, East, Elchlepp, & Monsell, 2019) have documented substantial preparation-related switch-repeat differences extending until (and even beyond) stimulus onset – this differential activity would severely contaminate a pre-stimulus baseline. We discarded segments associated with error trials, trials following errors, and the first trial of a block. The resulting segments were visually inspected for ocular, muscle, movement and other artifact and segments containing such artifact were removed. The remaining EEG segments were averaged for every participant and experimental condition. We conducted a detailed ERP analysis looking for a potential modulation (induced by task and task switches) of the amplitude, or latency, of the EEE.

*EEE amplitude*. Eimer and colleagues found the EEE to emerge from ~150 ms and extend for several hundred milliseconds (see Eimer and Holmes, 2007). We averaged the amplitude in three 100 ms time windows (150–250 ms, 250–350 ms, 350–450 ms). To assess potential interactions of the experimental conditions with spatial (scalp topography) dimensions, the amplitude was averaged for groups of electrodes along the anterior-posterior (anterior frontal, posterior frontal, parietal, occipital) and laterality (left, middle, right) dimensions, and submitted to ANOVAs for above time-windows with the factors switch (switch, repeat), task (face, letter), facial expression (emotional, neutral), region, and laterality. Significance levels were adjusted using the Huynh-Feldt correction for violations of sphericity (but unadjusted degrees of freedom are reported). To pre-empt, these analyses did not find any significant differences in the magnitude of the EEE between switches and repeats or between the face and letter tasks. To ensure that our whole-scalp analysis did not miss any topographically circumscribed effects, we also conducted analyses on an area of the scalp where the amplitude of the EEE was maximal. The early portion of the EEE has previously been found to have a fronto-central distribution; in our case the topography of the effect was also slightly left-lateralised. We therefore averaged amplitudes over electrodes AF1, F1, F3, F5, FC1, FC3, FC5, FCz, Fz and submitted them to switch × task × facial expression ANOVAs for the three time-windows.

**Figure 3:**
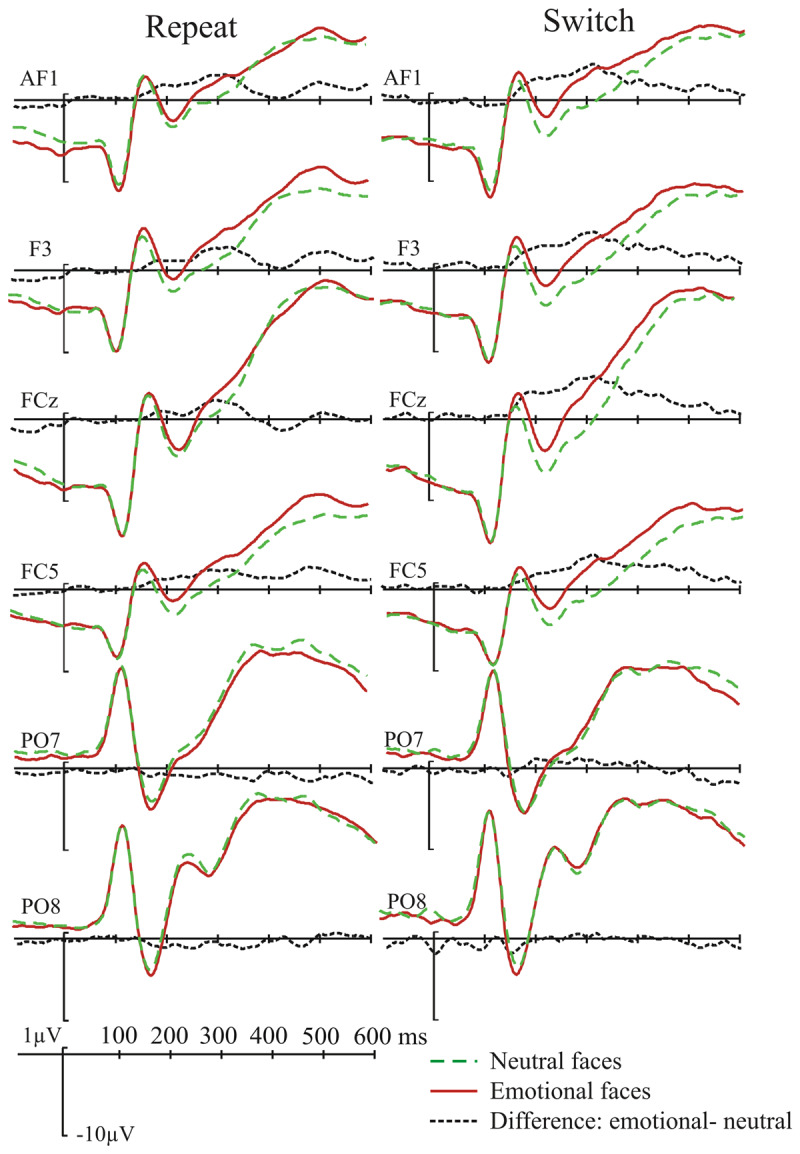
Grand-average ERPs for the face task as a function of emotional expression and switch/repeat. The electrodes were selected to illustrate both the Emotional Expression Effect (EEE, in frontal electrodes), or encompass the N170 peak in temporo-occipital electrodes.

*EEE latency*. A key aim was to determine whether the EEE was delayed on face task trials after a switch from the letter task relative to face task following the face task. Inspection of the EEE in our difference waves (see ***Figure 3***) revealed for both switches and repeats a broad peak, for which latency estimation can be problematic because even minor errors in determining maximal amplitude can result in large errors in the estimated peak latency. Moreover, the rising portion of the EEE was not sufficiently monotonic/linear to estimate the onset of the EEE using bilinear function fitting (as in Elchlepp et al., 2015). We therefore used a method developed by Elchlepp et al. (2017), which estimates the delay (temporal shift) of a portion of the difference wave, rather than a single point. The method uses the cross-correlation of difference waves corresponding to two experimental conditions (e.g., the EEE for switches vs. the EEE for repeats) as a measure of similarity of the two difference waves. We computed the bivariate Pearson correlation while ‘sliding’ one difference wave relative to the other, in 2 ms steps (given the sampling rate of 500 Hz), back in time and forward, and without any shift. The time-point at which the correlation coefficient is maximal indicates the temporal shift required to maximize the similarity between the two difference waves – and provides a measure of delay of the switch condition difference wave (relative to the repeat condition difference wave). To apply the procedure, one must select the portion of the difference waves corresponding to the EEE effect. Several options present themselves here: one can analyze a shorter or a longer segment; one can extract a segment corresponding to the rising part of the EEE, or centered around its peak, etc. To prevent such decisions influencing the outcome of the delay analysis, we conducted it for several segments of different lengths and positions within the difference wave (details in Results).

Individual participants’ ERPs (and the resulting difference waves) have a low signal-to-noise ratio (SNR). Hence, we used the ‘jackknifing’ method (Miller, Patterson & Ulrich, 1998) developed to maximize the SNR by computing the *t* statistic on the basis of averages over participants, rather than individual participants’ ERPs. This procedure uses grand-average waveforms of all but one participant leaving out a different participant each time – this was done for each combination of switch/repeat and emotional/neutral expression. We then computed for the switch and repeat conditions the emotional-minus-neutral difference waves and averaged over the electrodes where the EEE was maximal (see above section on amplitude analyses). Finally, we estimated the shift (delay) for each ‘jackknifing’ grand-average and submitted the resulting delay estimates to a *t* statistic to determine whether the delay was significantly different from zero, computing the standard error using Miller et al.’s (1998) formula.

## Results

### Behavioral results (see ***Figure 2***)

The first trial of a block, trials following errors and trials with RTs below 200 ms or above 3000 ms were excluded from the analysis. We were primarily interested in effects of task and a change of tasks after preparation for the cued task, i.e. at the long CSI. To ascertain that participants indeed used the preparation interval (CSI) effectively, we examined the reduction in switch cost with an increase in CSI. An ANOVA with the factors switch/repeat, CSI, task, and facial expression found that RT switch cost indeed reduced reliably from 77 ± 10 ms when the CSI was 200 ms to 50 ±8 ms when the CSI was 1000 ms; a reduction of 27 + 9 ms; for the switch × CSI interaction, *F*(1, 19) = 9.8, *p* = 0.005, η*_p_^2^* = 0.340. Error rates also reduced from 2.5% in the short to 1.7% in the long CSI, but this reduction was not significant (*p* = 0.15). The ‘residual’ switch cost (main effect of switch/repeat for the long CSI) was statistically significant for both RTs, *F*(1, 19) = 43.86, *p* < 0.001, η*_p_^2^* = 0.698, and errors, *F*(1, 19) = 60.33, *p* < 0.001, η*_p_^2^* = 0.760. Because our ERP (EEE) analyses below focus on the face task, we also examined the reduction in switch cost with preparation in the face task specifically. Here the switch cost reduced significantly, from 95 ± 13 ms in the short CSI to 43 ms ± 8 in the long CSI, a reduction of 52 ± 14 ms, *F*(1, 19) = 13.13, *p* = 0.002, η*_p_^2^* = 0.409. The residual switch cost was also significant, *F*(1, 19) = 26.66, *p* = 0.001, η*_p_^2^* = 0.469.

Since the ERP analyses were conducted for the long CSI trials only, we ran a further ANOVA for long CSI trials with the factors switch/repeat, task, and facial expression. It showed that responses were slower for switch (740 ms) than repeat (690 ms) trials, *F*(1, 19) = 43.86, *p* < 0.001, η*_p_^2^* = 0.698, and slower in the face (724 ms) compared to the letter task (706 ms), *F*(1, 19) = 5.42, *p* = 0.03, η*_p_^2^* = 0.222. For the face task, responses were faster for emotional (706 ms) than for neutral faces (741 ms), but the letter task showed the opposite pattern – faster responses for letters superimposed on neutral (691 ms) than for letters superimposed on emotional faces (721 ms); task by face expression interaction, *F*(1, 19) = 55.73, *p* < 0.001, η*_p_^2^* = 0.746. Error rates were higher for switch (4.6%) than repeat (2.9%) trials, *F*(1, 19) = 60.33, *p* < 0.001, η*_p_^2^* = 0.760, in the face task (5.1%) compared to the letter task (2.3%), *F*(1, 19) = 28.14, *p* < 0.001, η*_p_^2^* = 0.599, and for emotional (4.2%) compared to neutral faces (3.3%), *F*(1, 19) = 6.42, *p* 0.02, η*_p_^2^* = 0.252. The error switch cost was larger for the face (2.7%) than the letter task (0.6%), *F*(1, 19) = 12.06, *p* < 0.003, η*_p_^2^* = 0.388, and more errors were made for emotional than neutral faces in the face task (1.7%) compared to the letter task (0.1%), *F*(1, 19) = 4.55, *p* = 0.046, η*_p_^2^* = 0.193.

To determine whether expression influenced performance on the subsequent trial^4^ in either task on the long CSI trials (for which we had more substantial cell sizes), we performed an additional ANOVA with factors n-1 facial expression, task and switch/repeat. None of the interactions of interest (those involving the factor n-1 facial expression) approached significance. Finally, we also ran an ANOVA for the long CSI letter task trials with the factors facial expression, switch/repeat and the additional factor response congruency – which contrasts ‘congruent’ face-letter stimulus compounds where both elements of the stimulus were mapped on the same response with ‘incongruent’ stimuli, where the two elements were mapped on different responses. The aim of this analysis was to examine whether emotional faces resulted in greater activation of the irrelevant S-R rule in the letter task than neutral faces (the facial expression × congruency interaction, possibly with a further modulation by switch/repeat). There was such a trend in the RT data, where a congruency effect was present on letter task trials when the face had an emotional expression (incongruent, 733 ms, congruent, 709 ms), but not when the face expression was neutral (incongruent, 691 ms, congruent, 690 ms), however the facial expression × congruency interaction did not approach statistical significance (*F* < 1.5). The opposite effect was found for the error rates, where incongruency increased error rates less for letters superimposed on emotional faces (incongruent, 2.55%, congruent, 2.05%), than for letters superimposed on neutral faces (incongruent, 4.08%, congruent, 0.65%), and this interaction was statistically significant, *F*(1, 19) = 9.56, *p* = 0.006, η*_p_^2^* = 0.335. Congruency also interacted significantly with switch/repeat, *F*(1, 19) = 19.64, *p* < 0.001, η*_p_^2^* = 0.508, reflecting a larger congruency effect during switches to the letter task (incongruent, 4.45%, congruent, 1.05%), than during its repetitions (incongruent, 2.18%, congruent, 1.65%). The 3-way interaction did not approach significance.

To sum up, the substantial reduction in switch cost (both overall by 35%, and specifically in the face task by 55%) with the increase in the CSI shows that participants prepared for a task switch. On long CSI face task trials, responses were faster for emotional than for neutral faces, though at the cost of more errors for the former. On long CSI letter task trials, responses were slower with emotional faces, but also less affected by response conflict (incongruency), than with neutral faces; possibly because encountering an emotional face resulted in increased caution (an adjustment of the response criterion).

### ERP amplitude analyses

**Figure 4:**
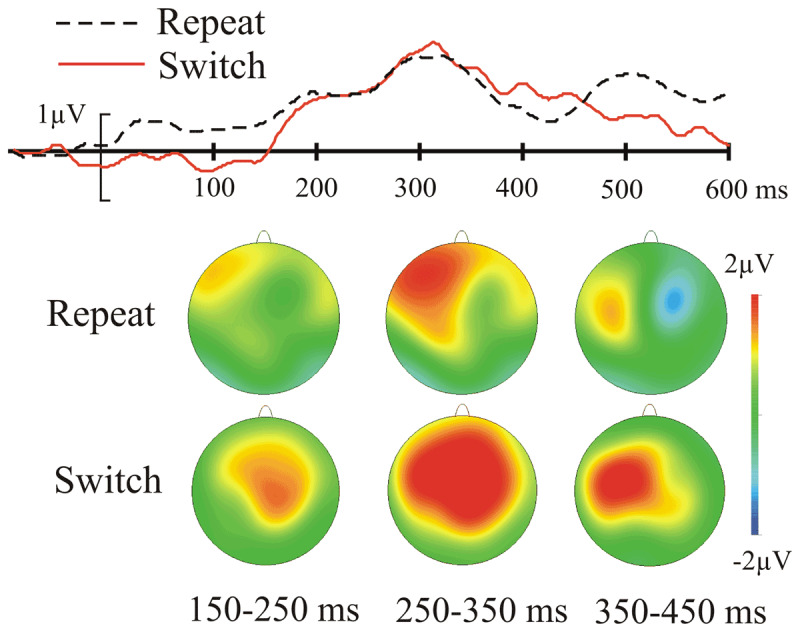
The emotional-neutral ERP difference (the EEE) for the face task as a function of switch/repeat: the upper panel shows the difference waves averaged for a subset of left frontal electrodes where the EEE is maximal (see Method); the lower panel shows the spline-interpolated scalp distribution of the EEE.

**Figure 5:**
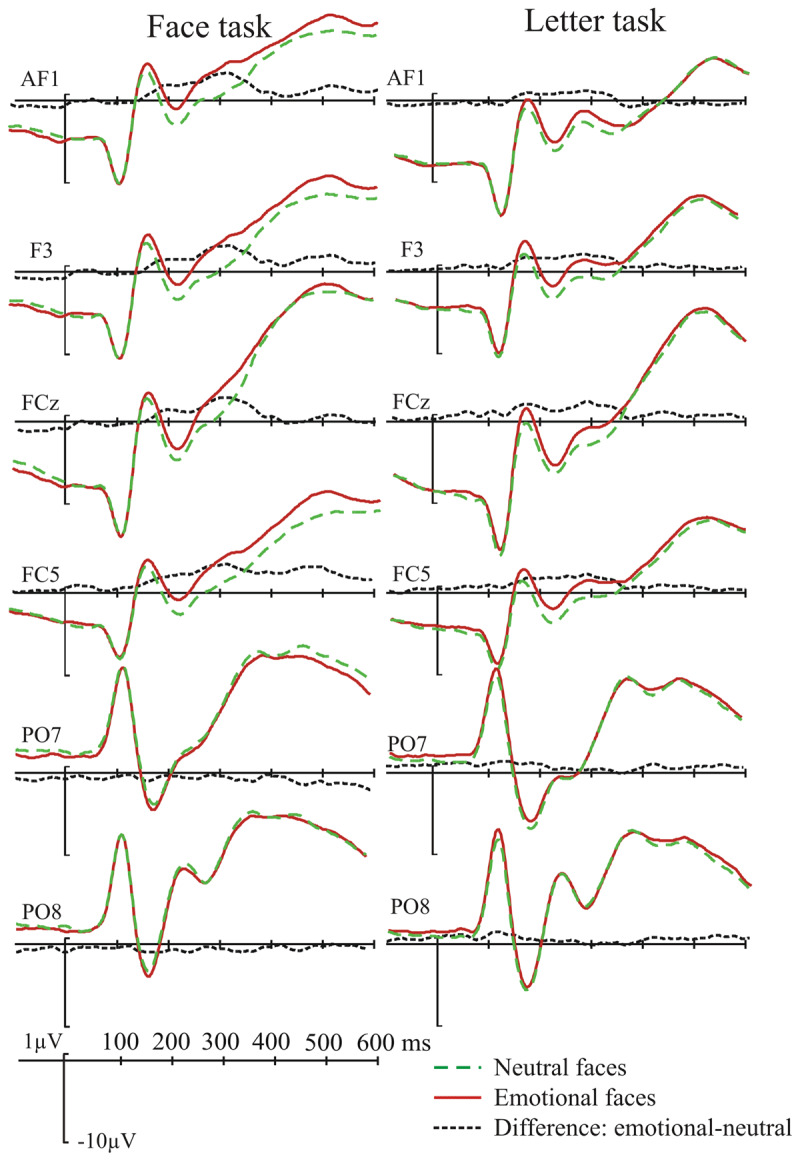
Grand-average ERPs as a function of emotional expression and task shown for the same set of electrodes as in Figure 3.

**Figure 6:**
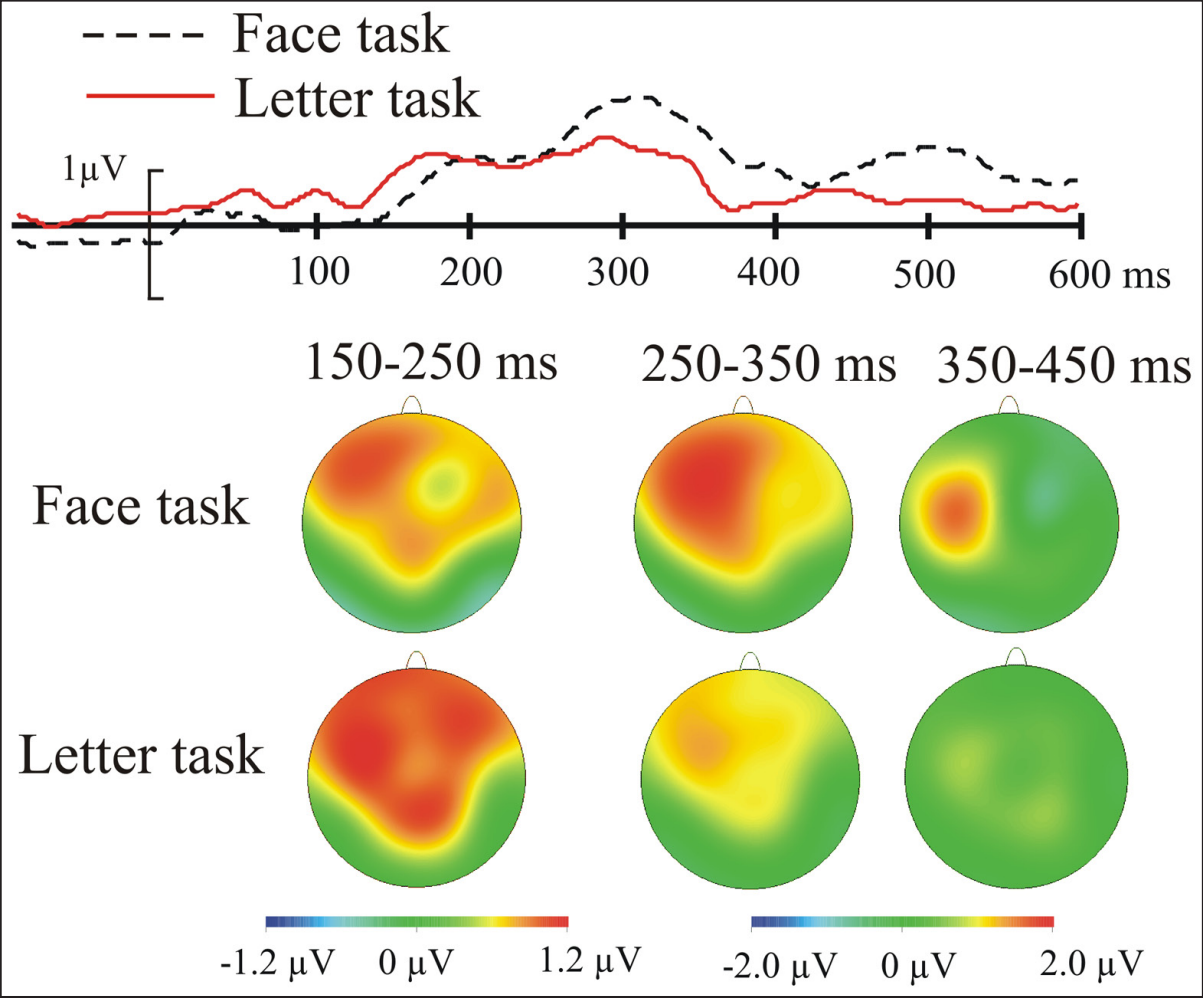
The emotional-neutral ERP difference (the EEE) for the two tasks. As in Figure 4, difference waves averaged for the left-frontal cluster of electrodes is displayed above the scalp distribution of the EEE.

As specified in the Method, ERP amplitude (presented in ***Figures 3*** and ***4*** for the switch vs. repeat comparison in the face task and ***Figures 5*** and ***6*** for the comparison between the face and the letter tasks) was submitted to switch/repeat × task × emotional expression × region × laterality ANOVAs for 3 time-windows (150–250 ms, 250–350 ms, 350–450 ms), separately.

There was a significant main effect of emotional expression at 150–250 ms: *F*(1, 19) = 9.02, *p* 0.007, η*_p_^2^* = 0.322, and at 250–350 ms: *F*(1, 19) = 15.38, *p* = 0.001, η*_p_^2^* = 0.447; with more positive amplitudes for emotional than neutral faces – the EEE. Emotional expression interacted significantly with the spatial factors region (150–250 ms: *F*(3, 57) = 7.6, *p* = 0.004, η*_p_^2^* = 0.268, 250–350 ms: *F*(3, 57) = 17.14, *p* = 0.001, η*_p_^2^* = 0.474), laterality (250–350 ms: *F*(2, 38) = 5.41, *p* = 0.009, η*_p_^2^* = 0.221) and region and laterality (150–250 ms: *F*(6, 114) = 2.77, *p* = 0.036, η*_p_^2^* = 0.127, 250–350 ms: *F*(6, 114) = 3.26, *p* = 0.013, η*_p_^2^* = 0.147), indicating that the EEE varied over scalp regions; it was maximal in the fronto-central regions (see ***Figures 4*** and ***6***).

In the 3^rd^ time-window (350–450 ms) the EEE appeared more confined to the left lateralised regions – there was no main effect of emotional expression, but it interacted significantly with laterality, *F*(2, 38) = 3.72, *p* = 0.044, η*_p_^2^* = 0.164, and region and laterality, *F*(6, 114) = 6.34, *p* < 0.001, η*_p_^2^* = 0.250. The key interactions between switch/repeat and emotional expression, *F*(1, 19) = 0.45, *p* = 0.5, η*_p_^2^* = 0.023, and emotional expression and task, *F*(1, 19) = 0.58, *p* = 0.8, η*_p_^2^* = 0.003 did not approach significance.

There was a main effect of switch/repeat at 250–350 ms: *F*(1, 19) = 10.7, *p* = 0.004, η*_p_^2^* = 0.361, and at 350–450 ms: *F*(1, 19) = 9.83, *p* = 0.003, η*_p_^2^* = 0.341. In all three time-windows switch/repeat interacted significantly with laterality, *F*(2, 38) = 4.3, *p* = 0.032, η*_p_^2^* = 0.186, *F*(2, 38) = 9.3, *p* = 0.001, η*_p_^2^* = 0.328, *F*(2, 38) = 9.98, *p* < 0.01, η*_p_^2^* = 0.344. In the first two time-windows switch/repeat interacted with task and laterality, *F*(2, 38) = 4.1, *p* = 0.029, η*_p_^2^* = 0.176, *F*(2, 38) = 3.7, *p* = 0.033, η*_p_^2^* = 0.165. Because the factor emotional expression (the focus our hypotheses) was not involved in these interactions, they were not followed up with extra analyses.

In addition to the whole-scalp analysis above, we conducted a more spatially-specific analysis on the scalp region where the EEE was maximal (see Method for the relevant electrode grouping) – indeed one cannot conclude there is no significant modulation of the EEE by switch/repeat and task unless one takes all the steps to maximise the sensitivity of the analysis. A switch/repeat × task × emotional expression ANOVA for this region revealed, as in the whole scalp analysis, a significant EEE for the first two time-windows, *F*(1,19) = 14.9, *p* = 0.001, η*_p_^2^* = 0.440, *F*(1, 19) = 23.32, *p* < 0.001, η*_p_^2^* = 0.551, respectively, but no significant interactions in these two time-windows with switch/repeat, *F*(1, 19) = 0.02, *p* = 0.9, η*_p_^2^* = 0.001, *F*(1, 19) = 0.7, *p* = 0.4, η*_p_^2^* = 0.035, or task, *F*(1, 19) = 0.00, *p* = 0.99, η*_p_^2^* = .000, *F*(1, 19) = 1.67, *p* = 0.2, η*_p_^2^* = 0.080. In the 3^rd^ time-window, where the main effect of emotional expression failed to reach significance, *F*(1, 19) = 3.29, *p* = 0.09, η*_p_^2^* = 0.148, the interactions with switch/repeat, *F*(1, 19) = 1.53, *p* = 0.2, η*_p_^2^* = .076, and task, *F*(1, 19) = 0.82, *p* = 0.4, η*_p_^2^* = 0.041, were also non-significant.

To examine whether there is statistical support for the absence of a difference in EEE amplitude for the face task vs. the letter task and for switches vs. repeats in the face task, we computed Bayes Factors (BFs) by submitting the emotional-neutral difference in this scalp region to Rouder, Speckman, Sun, Morey & Iverson’s (2009) calculator (*http://pcl.missouri.edu/bf-one-sample*, see ***Table 1*** in Wetzels, Matzke, Lee, Rouder, Iverson, & Wagenmakers, 2011, for a detailed interpretation of Bayes Factors) for the first two time-windows, where a clear EEE was found. This resulted for the 150–250 ms time-window in a BF of 0.23 for the EEE amplitude comparison in the two tasks, and a BF of 0.27 for comparing the EEE amplitude for the switches vs. repeats – substantial evidence favouring the null hypothesis in both comparisons. For the 250–350 ms time-window, the BF of 0.48 for the comparison of the EEE amplitude in the two tasks and the BF of 0.32 for comparing the EEE amplitude for the switches vs. repeats, provided anecdotal-to-substantial evidence for the null.

**Table 1:** Descriptive and inferential statistics for the analysis of the switch-induced delay on the face task trials, with segments extracted from the difference wave based on the time-windows used for the amplitude analyses.


WINDOW WIDTH (START, END) IN MS	SHIFTS (LEFT, RIGHT) IN STEPS OF 2 MS	SHIFT WITH HIGHEST CORRELATION IN MS	T (19)	P	95% CONFIDENCE INTERVALS	BAYES FACTOR

100 (150–250)	30	2	0.05	0.96	[39.4, –37.4]	0.23

100 (250–350)	30	–2	–0.15	0.88	[12.3, –14.3]	0.23

200 (150–350)	60	2	0.2	0.84	[10.7, –8.7]	0.24


To summarise, at 150–350 ms a significant EEE was found in both tasks, and for both switch and repeat trials. There was no sign of a significant reduction in its magnitude in the letter task or during a switch from the letter to the face task. Importantly, no modulations of the EEE by switch or task were found even when testing only the scalp region where EEE amplitudes were maximal. In the 350–450 ms time-window the amplitude of the EEE decreased and it was no longer statistically significant – which was true for switches and repeats, and for both tasks.

### ERP latency analyses

For the analysis of a possible switch-induced delay in the EEE in the face task, we first extracted two 100 ms segments from the ERP difference waves corresponding to the first two time-windows used for the amplitude analyses above (150–250 ms and 250–350 ms); we also extracted a longer segment (200 ms) comprising both time-windows. We did not analyse the portion corresponding to the third (350–450 ms) time-window, because the EEE was decreased and barely detectable (non-significant) in this interval (see above amplitude analyses). The results from these analyses are summarised in ***Table 1***. The t tests comparing the delays, which ranged from –2 ms to 2 ms, found no evidence of a delay induced by a task-switch; the Bayesian statistics found substantial evidence for the null (no shift for switch versus repeat) for all three segments. It could be argued that the segments for the delay analysis should not be selected on the basis of the amplitude analysis, but based on the EEE morphology to capture the most characteristic portion of the effect. We therefore conducted an additional delay analysis for which the extracted segments were centred around the peak of the EEE. Four such segments of increasing length (from 150 to 300 ms in steps of 50 ms) were submitted to the analysis. The relevant statistics are presented in ***Table 2***. As with the previous analysis, the t statistic found no evidence that the mean delay of 4–10 ms was anywhere near statistical significance and Bayesian statistics found substantial support for the absence of a delay.

**Table 2:** Descriptive and inferential statistics for the analysis of the switch-induced delay on the face task trials, with segments centred around the peak of the EEE.


WINDOW WIDTH (START, END) IN MS	SHIFTS (LEFT, RIGHT) IN STEPS OF 2 MS	SHIFT WITH HIGHEST CORRELATION (MS)	T (19)	P	CONFIDENCE INTERVALS	BAYES FACTOR

150 (224–374)	50	4	0.33	0.75	[13.9, –9.9]	0.24

200 (200–400)	60	4	0.29	0.77	[15.5, –11.5]	0.24

250 (276–426)	70	8	0.7	0.5	[15.3, –7.3]	0.29

300 (150–450)	80	10	0.82	0.4	[16.9, –6.9]	0.32


### ERPs: vowel-consonant differences

**Figure 7:**
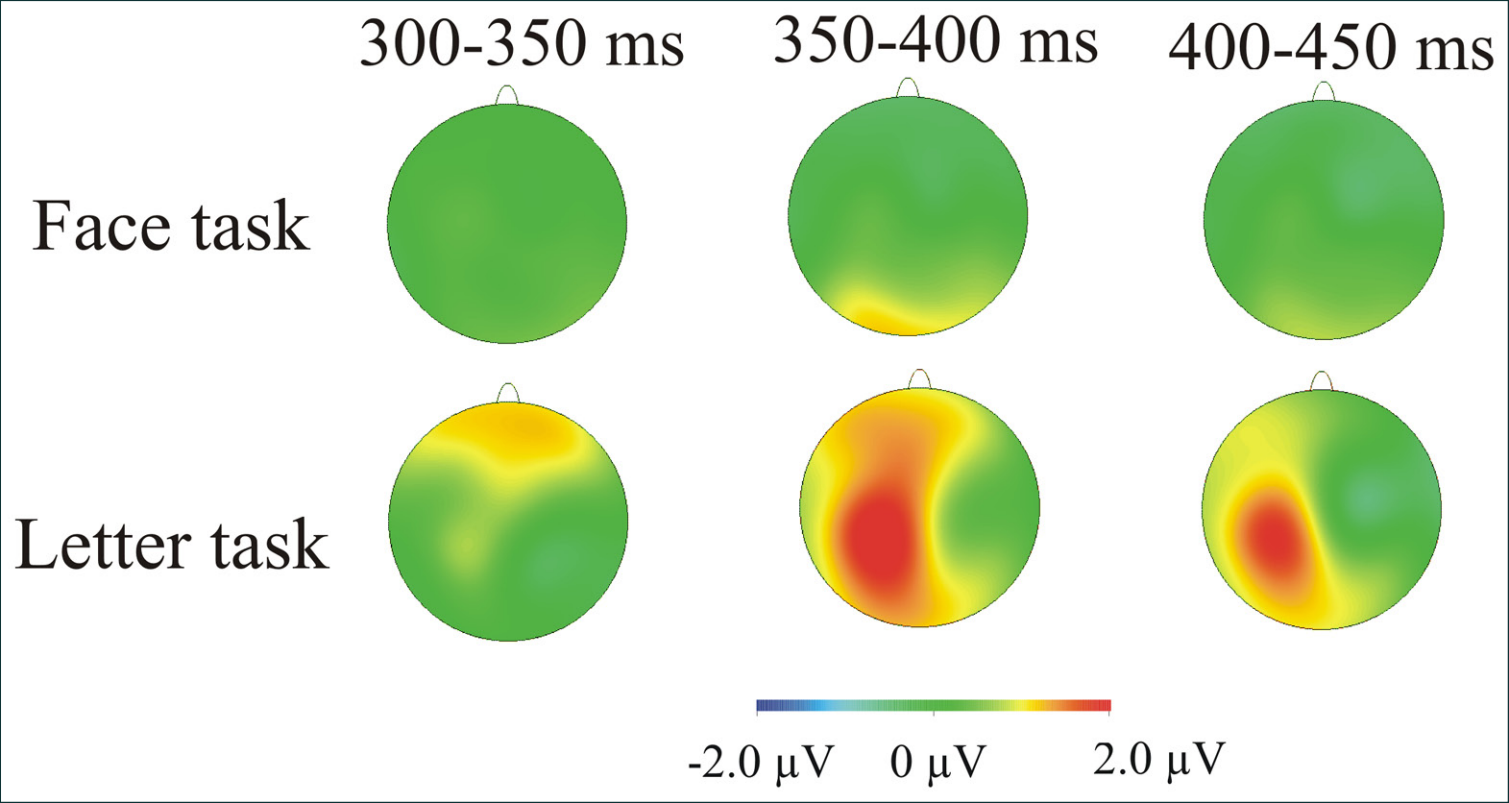
The scalp distribution of the vowel-consonant ERP difference as a function of task.

We had no *a priori* reason for expecting ERP differences between vowels and consonants. But if such differences were present, their interaction with switch and task could reveal if, unlike emotional expression, the encoding of letter form is subject to task-set influences, as suggested by recent research (Elchlepp et al., 2015). The inspection of the ERPs revealed an amplitude difference between the vowel and consonant trials (see ***Figure 7***), starting from about 300 ms and extending for ~150 ms. We sub-divided this stretch of ERP (300–450 ms) into three 50-ms time-windows and submitted each time-window to an ANOVA with the factors vowel/consonant, switch, task, region and laterality. No interactions involving switch and vowel/consonant approached significance in any of the three time-windows – but there were interactions involving vowel/consonant and task, along with region or laterality.

For the 300–350 ms time-window, the significant vowel/consonant × task × region, interaction, *F*(3, 57) = 7.71, *p* = 0.003, η*_p_^2^* = 0.289, was followed by separate analyses for the two tasks. In the letter task, vowel/consonant interacted with region, *F*(3, 57) = 9.83, *p* = 0.001, η*_p_^2^* = 0.341; testing the four regions revealed a significant main effect of vowel/consonant in the frontal anterior region, *F*(1, 19) = 8.0, *p* = 0.04, η*_p_^2^* = 0.296 (Bonferroni-corrected for multiple comparisons). There were no significant effects of vowel/consonant in the face task. In the following two time-windows, vowel/consonant interacted significantly with task and laterality, *F*(2, 38) = 4.37, *p* = 0.025, η*_p_^2^* = 0.187 (350–400 ms), *F*(2, 38) = 6.2, *p* = 0.005, η*_p_^2^* = 0.246, (400–450 ms). Analyses by task in the 350–400 ms time-window revealed robust and wide-spread vowel-consonant differences in the letter task (main effect of vowel/consonant, *F*(1, 19) = 5.1, *p* = 0.036, η*_p_^2^* = 0.211); in the face task the significant vowel/consonant by region interaction, *F*(3,57) = 8.46, *p* = 0.001, η*_p_^2^* = 0.308, was followed by non-significant effects of vowel/consonant in analyses by scalp region. Analyses by task in the 400–450 ms time-window found that vowel/consonant interacted significantly with laterality in the letter task (but there were no significant effects of vowel/consonant in the follow-up analyses by laterality), and with region in the face task (but no significant vowel/consonant effects in the follow-up analyses by region).

To summarise, ERP vowel-consonant differences were robustly modulated by task-set (see ***Figure 7***): they were significant only in the letter task (over the frontal scalp at 300–350 ms and more spatially widespread at 350–400 ms). Switching to the letter task did not significantly attenuate the amplitude of these effects relative to repeating this task.

## Discussion

Our review in Part 1 of a growing corpus of behavioural, EEG and eye-tracking studies indicated that when a change of cognitive task requires a shift of attention to another perceptual attribute (location, visual dimension, voice, lexical versus perceptual attribute), it usually prolongs/delays/limits the encoding of that perceptual attribute, even after a substantial interval for preparation. We have attributed this largely to ‘attentional inertia’ (Longman et al., 2013; 2014) – incomplete readjustment of attentional selection on a switch trial, when the relevant perceptual attribute changes, even after substantial time for preparation. In Part 2, we applied this methodology to another perceptual process – the encoding of facial emotional expression – which conveniently has a well-documented brain-potential correlate, the EEE (Eimer & Holmes, 2007). Our detailed amplitude and latency analyses indicated that switching from the task of categorising the letter to categorising facial expression produced neither a delay in the onset or evolution of the EEE, nor a reduction in its amplitude. This outcome is in marked contrast to the modulation of early stages of visual word recognition and colour processing by a task switch we have previously demonstrated (Elchlepp et al., 2015; 2017, see Part 1).

Why are shifts of attention to foveal facial expression special – with early stages of processing apparently impervious to ‘attentional inertia’? It is likely that with massive exposure, possibly primed by innate templates, each individual develops extremely efficacious perceptual representations (“attentional templates”) for the facial expressions of key emotions, which ensures the rapid and effective activation of these templates. Indeed Eimer and Kiss’s (2008) finding, reviewed in the Introduction, of the EEE being elicited in the absence of conscious awareness is consistent with this account. (See also Palermo & Rhodes, 2007, for a review of neuroimaging evidence).

A different (though not incompatible) type of account is in terms of prioritisation during perception. It may be that perceptual encoding of a face (whatever its expression) gets priority early during perception, or that any stimulus with a strong emotional connotation is prioritised, or a combination of the two – a face expressing emotion gets priority. Our ERP results cannot distinguish between these possibilities, but our RT findings make the first an unlikely account of our results. On letter task trials, emotional expressions prolonged RTs (relative to neutral expressions), but reduced the tendency to make more errors on response incongruent trials than on response congruent trials. This suggests that rapid detection of fear and anger (the two emotional expressions we used) may have increased response caution on letter task trials. Importantly, this did not reflect generally slower responses to emotional faces; in the face task the pattern was reversed: responses were faster for emotional faces than for neutral faces. A similar influence of emotional expression in the non-emotion task was recently reported by Berger, Richards, and Davelaar (2019) who asked participants to alternate (in runs of two trials) between two categorisations: ‘happy’ vs. ‘neutral’ and ‘young’ vs. ‘old’. They found longer RTs on age categorisation trials (for both switches to and repetitions of this task) when the face was happy than when it was neutral. Our results and those of Berger et al. (2019) appear to be inconsistent with those of Schuch, Werheid and Koch (2012), who required participants to switch between two of three categorisations of faces (by emotional expression, gender and age) and found that the effect of response congruency (longer RTs for response-incongruent trials – a measure of interference from the competing set of S-R mappings) was greater in the emotional expression task than in the gender and age tasks;^5^ there was no significant response congruency effect in the gender and age tasks when participants switched between either of them and the emotional expression task. However, we note that this study did not compare the effect of an emotional face stimulus to that of a non-emotional (‘neutral’) face stimulus on performance in the non-emotion tasks. Furthermore, the use of non-cropped images of faces meant that participants in the age and gender tasks could direct their spatial attention to non-facial features (hair length and colour, size and shape of neck and head) thus reducing the interference from facial expression.

There is some evidence that emotion-driven prioritisation in the context of task-switching may not be confined to faces. Paulitzki, Risko, Oakman and Stolz (2008) presented participants with digits superimposed on colour photographs of spiders and asked them to alternate (in runs of two trials) between classifying the texture of the spider (hairy vs. smooth) and the parity of the digit; a visual word (reminder) cue (“Spider-Texture” or “Odd/Even”) was presented on each trial at a CSI of 1000 ms. There was a smaller switch cost in the spider task than in the digit task, and most of this interaction appeared to come from the switch trials, whose RTs were shorter and error rates lower on spider task trials. Correlational analyses showed that questionnaire-assessed fear of spiders predicted lower error rates on switches to the spider task and higher error rates when switching away from it (to the parity task). The authors interpreted these results as suggesting that their spider photos were associated with ‘accelerated engagement’ and ‘decelerated disengagement’.

Our investigation of the influence of task-set on the recognition of facial emotional expression also has implications concerning its “automaticity”. In Part 1 we distinguished two aspects of automaticity – involuntariness and invariance – and in the introduction to Part 2, we reviewed Holmes et al.’s (2006) use of the EEE to examine the involuntariness and invariance of discriminating fearful and neutral facial expressions. Their key finding was that the early portion of the EEE during the line judgement task (where faces were irrelevant) was statistically indistinguishable from the face judgement task (detecting repetitions of a face). Although Holmes et al.’s finding suggests that the recognition of fear is both involuntary and (in its early stages) invariant, there are at least two plausible alternative interpretations. First, in the line judgement task faces were presented centrally, whereas the two lines were presented on either side (at 4⁰° eccentricity), so the face was the first (and possibly only) stimulus element to be perceived foveally. Thus, it is possible that the robust early portion of the EEE in the line task was a reflection of the processing “boost” conferred by foveation. Another alternative interpretation is that although non-spatial attentional selection is capable of suppressing the encoding of facial expression, participants did not need to use (effortful) non-spatial selection. They could rely entirely on allocating the distribution of spatial attention to the predictable locations of the lines. This would account for the presence of the EEE up to a point when spatial attention was allocated to the lines, at which point the EEE was abolished.

Our study has the potential to decide between these interpretations. First, both elements of our stimulus compound were presented centrally, which ensured that the letter and the eyes-nose-mouth region of the face were simultaneously foveated. Second, three characteristics of our stimuli made it very unlikely that participants could use spatial attention alone to select between the regions of the face diagnostic of emotional expression and those diagnostic of letter identity: (1) these regions overlapped substantially, (2) the letter was transparent (except for a thin outline) so that the face content remained clearly visible, and (3) the letter changed in size and shape over trials, making it difficult to configure a suitable spatial attention ‘spotlight’ to select either set of regions.

Hence, non-spatial attention (to face versus letter features) was essential to select the task-relevant perceptual attribute(s) in the stimulus. This non-spatial perceptual biasing was clearly effective in controlling processing: participants responded correctly and with low error rates. But at the same time the ERP index of facial expression recognition – the EEE – was by two measures unaffected by the task (attentional) set. First, a change from the vowel-consonant task to the emotional vs. neutral task did not delay or reduce the amplitude of the EEE. One might argue that an effect of task switch on EEE could have been found for short CSI (“unprepared”) switches to the face task – which we did not analyse, because we included only a small number of such trials to maximise power for the long CSI condition, in which we were primarily interested (see Introduction and Method).^6^ However, we note that our previous evidence of a task-switch prolonging perceptual encoding of colour and word form was obtained in conditions of “prepared” switches with a comparable CSI (800 ms in Elchlepp et al., 2015; 1000 ms in Elchlepp et al., 2017). Perhaps more striking is our second measure of independence of EEE from task (attentional set): the EEE amplitude was numerically and statistically indistinguishable in the two tasks for about the first half of its time-course (and the numerical differences in the second half did not approach significance), despite the potential conflict from the S-R mappings of the (irrelevant) face task presumably serving as a strong incentive to suppress the encoding of the face’s perceptual attributes. This is in striking contrast with the previously documented effects of task-set on ERP indicators of lexical processing (e.g., Elchlepp et al., 2015; Kiefer & Martens, 2010; Vachon & Jolicoeur, 2011, 2012) – these indicators were strongly attenuated in non-lexical tasks, even when the task ensured the processing of the whole string (Elchlepp et al., 2015). The EEE results would also appear to contrast with the observation in the present study of vowel-consonant ERP differences whose amplitude was clearly modulated by task-set (though not by a task switch). Indeed, the vowel-consonant differences present in the letter classification task were strongly attenuated (and statistically non-significant) in the face task.

Could it be the case that influences of task (attentional) set on ERP effects such as the EEE are just generally modest and require more statistical power to be detected. Our Bayes Factors were indeed mostly in the 0.2–0.3 range, providing substantial, but not strong or very strong, evidence for H_0_ (according to the Wetzels et al.’s, 2011, classification). We can examine this from two perspectives.^7^ First, we can compare our number of participants and trials per participant to those in Holmes et al. (2003), who found that endogenous shifting of spatial attention away from non-foveal faces abolished the EEE. Our study had slightly more participants (20 vs. 18), but nearly 40% more trials per participant (920 vs. 680), hence it was better powered, containing ~50% more observations overall. Second, we can compare the effect size (Cohen’s d) for the statistically non-significant effect of task set on the EEE in the current study (in the left fronto-central region where the EEE was maximal) with the statistically significant effect of task set we observed on the ERP marker of lexical access – the ERP difference between words with a high lexical frequency and words with a low lexical frequency (Elchlepp et al., 2015, see Part 1): d_v_ = 0.03 and d_v_ = 0.84 respectively. Thus, the effect size for the currently reported influence of task set on EEE is more than an order of magnitude smaller than the effect size for the previously documented influence of task set on the ERP lexical frequency marker.

One caveat to note is that in our face perception task expression was relevant with respect to the response categories – emotional and neutral expressions were mapped onto the two responses in the face task. In the study by Elchlepp et al. (2017), which showed that switching from the letter task prolonged colour processing, the ERP contrast based on the frequency (probability) of colour presentation was orthogonal (incidental) to the required ‘warm/cold’ colour classification (the manipulation of colour probability was also not mentioned to participants before or during the experiment). Similarly, in Elchlepp et al.’s (2015) Experiment 1, which showed that the ERP effect of word frequency was delayed by a switch from the perceptual (colour symmetry) task to the lexical task and strongly attenuated in the perceptual task, the lexical frequency contrast was orthogonal to the semantic categorisation task (and it was not mentioned to participants until the post-experiment debriefing). Hence, an important question is whether early encoding of the emotional expression would also be impervious to task-set when emotional expression is orthogonal (and incidental) to all task-relevant categories (and sets of S-R mappings). In the study by Holmes et al. (2006) discussed earlier this appeared to be the case: the EEE was found in the condition where participants had to attend to the identity of the face and detect repetitions of the same individual irrespective of expression. However, detecting repetitions of the same person over different facial expressions may require some encoding of expression to separate intra-individual from inter-individual variability (to ensure that different expressions of the same face are not encoded as different individuals).

Several more recent studies (Itier & Neath-Tavares, 2017; Neath-Tavares & Itier, 2016; Rellecke, Sommer, & Schacht, 2012) have addressed this issue by comparing the ERP effects of facial expression in tasks requiring emotion discrimination to those in tasks where emotion was irrelevant and did not involve the identification of a person’s face (across different expressions). In all these studies the emotional versus neutral contrast revealed in the emotion recognition task robust and protracted ERP effects with very similar topography to the EEE – an emotion-related positivity in fronto-central regions of the scalp accompanied by a negativity in posterior regions of the scalp (though the authors tended to select mostly posterior electrodes for statistical analyses).^8^ In addition to the emotion recognition task, Rellecke and colleagues (2012) presented angry, happy and neutral faces in a gender discrimination task and (intermixed with words) in another task requiring categorisation as face vs. word. In both of these tasks the angry-neutral ERP difference at 0–300 ms following face onset was statistically indistinguishable from the effect in the emotion recognition task. The happy-neutral difference was reduced in the face/word categorisation – though it seems unlikely that the required perceptual discrimination in this task ensured adequate allocation of spatial attention to the eyes-nose-mouth region of the face, which is critical for the emergence of ERP effects of emotional expression (Holmes et al., 2003, see Introduction). Neath-Tavares and Itier (2016) and Itier and Neath-Tavares (2017) presented faces with fearful, happy and neutral expressions in an emotion recognition task and in an odd-ball task requiring detection of flowers presented instead of faces on 20% of trials. Itier and Neath-Tavares (2017) also used gender classification.^9^ The results showed that the robust effects of expression on ERPs were no smaller in the gender and odd-ball tasks than in the emotion discrimination task (no statistical interactions with task).

Thus, the outcomes of these three studies (Itier & Neath-Tavares, 2017; Neath-Tavares & Itier, 2016; Rellecke et al., 2012) are consistent with our conclusion that the perceptual encoding of emotional expression up to ~300 from the onset of a face is both involuntary and invariant. However, it can be argued that, because in these studies faces were presented on their own, the perceptual encoding of expression may have proceeded in parallel (and without interference) with the encoding of task-relevant (e.g., gender) information. Thus, it is important to determine to what extent facial expressions (not mapped to any responses) are encoded when faces overlap foveally with task-relevant non-face stimuli. Müller-Bardorff and colleagues (Müller-Bardorff, Schulz, Peterburs, Bruchmann, Mothes-Lasch, Miltner, & Straube, 2016) did just that by asking participants to compare the length of two horizontal lines superimposed (positioned below the eyes) on task-irrelevant angry, happy and neutral faces. The authors also manipulated the intensity (high vs. low) of the emotional expression and the perceptual demands (high vs. low) of the line task. Unfortunately, given the small spatial extent of the lines (relative to the eyes-nose-mouth region of the face), it seems likely that participants could focus spatial attention (see above and the Introduction), ‘zooming in’ to the lines to reduce the encoding of the nose and mouth regions. The results showed that the intensity of emotional expression modulated ERP amplitudes irrespective of task demand, consistent with the invariance of encoding emotional expression. Unfortunately, the authors did not explicitly test for the reduction in the angry-neutral difference and the happy-neutral difference in the high-demand relative to the low-demand condition of the line task, which would have enabled firmer conclusions regarding the invariance of expression effects with respect to the demands of the non-face task. Hence, further investigations (with improved control for the spread of spatial attention) will be needed to determine whether the processing of non-face stimuli that overlap foveally with task-irrelevant faces influences the encoding of emotional expressions which are not mapped to responses.

We conclude that attentional selection of visual features does little to prevent the perceptual encoding of facial expression when it is not (or no longer) task-relevant. In the context of the influences of task-set on the perceptual encoding of other kinds of stimuli, in particular the task/attention-switching studies reviewed in Part 1, this strongly supports the view that the detection of facial emotional expression is a special case of independence from the effects of top-down task (attentional) set. More broadly, we hope we have illustrated, with both a general review and a detailed example, how switching paradigms developed to study an important aspect of multi-tasking can shed light on the potential contribution of attentional inertia to the costs of switching between tasks, and on the degree of automaticity of different kinds of perceptual process.

## Data Accessibility Statement

The data are available on the UK data service Reshare, *http://doi.org/10.5255/UKDA-SN-855092*.

## Footnotes

Multiple criteria for whether a process is ‘automatic’ have been considered, including: happens without awareness of the stimulus; does not require central capacity or resources that can be flexibly allocated between tasks; happens without, and in spite of, intention. We limit our consideration to the last of this triad (involuntariness), and thus do not consider evidence on awareness (as assessed by subliminal presentation), or on the central capacity requirement – e.g. as assessed by the PRP paradigm (e.g. Lien et al, 2008).

In a second experiment Vachon and Jolicoeur (2012) compared a condition in which both stimuli were classified by whether they were related to the context word, and one in which the first stimulus was classified as living/non-living – two semantic decisions. Marked attenuation of N400 was again observed in the latter case. Clearly there is no shift in spatial attention required here. But the N400 suppression here may derive from the rapid shift in S-R rules required.

The recognition of digits is at least as involuntary as the recognition of words; for example, the RT for counting digits is harder when their value conflicts with their numerosity (Morton, 1969).

We thank a reviewer for suggesting this analysis and the following analysis examining the interaction of facial expression and congruency in the letter task.

Schuch et al. (2012) also found greater switch costs and ‘mixing costs’ in the gender and age categorisation tasks when participants switched between them than when participants switched between either of these tasks and the task of categorising emotional expression.

We thank a reviewer for making this point.

We thank a reviewer for raising this possibility and for suggesting the two approaches below for evaluating it.

These studies, unlike ours and those of Holmes, Eimer and colleagues, re-referenced their EEG data to an average (common) reference before their ERP analysis. This can change the “local” amplitude of the effect within circumscribed scalp regions – a shift in the balance of the positive vs. negative poles of the scalp distribution. For example, the effect of re-referencing our EEE to the average reference would be to increase the posterior emotion-related negativity and reduce the fronto-central positivity.

A further key manipulation in both studies was a gaze-contingent positioning of the face on the display – which ensured that eye fixation was on a specific feature of the face (the left eye, the right eye, the nose, the mouth). We do not review the ERP correlates of this manipulation because they were not modulated by the task × expression interaction, in which we are interested.
